# Supplementary material for: Single cohesin molecules generate force by two distinct mechanisms
Source: Nat Commun. 2023 Jul 4;14:3946. doi: 10.1038/s41467-023-39696-8 (PMC10319895; doi:10.1038/s41467-023-39696-8)
Supplement: Supplementary file 1 — Supplementary Information [file 41467_2023_39696_MOESM1_ESM.pdf]

Supplementary Information

Single cohesin molecules generate force by two distinct mechanisms

Georgii Pobegalov[1,2], Lee-Ya Chu[1], Jan-Michael Peters[2] and Maxim I. Molodtsov[1,2,3,*]

[1] The Francis Crick Institute, London, NW1 1AT, United Kingdom

[2] Department of Physics and Astronomy, University College London, London, WC1E 6BT, United Kingdom

[3] Research Institute of Molecular Pathology (IMP), Vienna BioCenter, Vienna, 1030, Austria

[*] For correspondence:

m.molodtsov@ucl.ac.uk (MM)

This file contains:

Supplementary Methods

Supplementary Figures 1 – 7

Supplementary methods

DNA loop extrusion assay

To make sure that human Biotin-HaloLigand cohesin coupled to a HaloTag Myosin coiled-coil handle is able to perform DNA loop extrusion, we verified it using a single-molecule assay as previously described [1] with minor changes (Supplementary Fig. 7c). The flow cell was first incubated with 30 µL of anti-Digoxigenin antibodies (Roche, 150U) diluted 1:30 in PBS for 15 min, followed by a 200 µL wash with PBS. The surface was further passivated by 10 min incubation with Pluronic F127 (Sigma-Aldrich, 1% solution in PBS), followed by a 200 µL wash with PBS and at least 1 hr incubation with β-Casein (Sigma-Aldrich, 10 mg/ml in PBS). Subsequently the flow cell was equilibrated with 40 µl of buffer D (0.1 mg/ml β-Casein in PBS). λ-phage DNA was end-labelled as previously described [1]. 40 µl of 10 pM of Digoxigenin-labelled λ-phage DNA in buffer D were introduced into the flow cell at 5 µl/min, incubated for 10 min and washed with 40 µl of buffer D at 5 µl/min. The flow cell was further equilibrated with 40 µl of buffer L (40 mM Tris-HCl pH 7.5, 50 mM aCl, 2 mM MgCl2, 5 mM ATP, 10 mM DTT, 250 nM Sytox Orange, 0.05 mg/mL BSA, 0.1 mg/mL β-Casein, 0.2 mg/ml glucose oxidase, 35 µg/ml catalase and 4.5 mg/ml dextrose) at 15 µl/min.

Human cohesin and a CC-handle were mixed in 150 µl of buffer R at 0.3 nM and 50 nM concentration respectively and incubated for 5 min at room temperature. Subsequently human NIPBL was added to a final concentration of 3 nM and the mixture was supplied to the flow-cell at 15 µl/min. Images of flow-stretched DNA molecules stained with Sytox Orange were recorded at 1 Hz using the JPK Nanotracker 2 equipped with a EMCCD camera (iXon Ultra 897, Andor). Fluorescent excitation was realized through a custom-built HILO microscopy set-up utilizing a 561 nm laser and a Nikon SR HP Apo TIRF 100x/1.49 oil immersion objective. Images were saved as uncompressed TIFF files and further processed using FIJI ImageJ. After 7 min of incubation with cohesin, NIPBL and ATP 56 % of DNA molecules (75 of 135) exhibited loops. All experiments were performed at room temperature.

To verify the coupling of the CC-handle to loop-extruding cohesin, we labelled CC-handle with Qdot 705 streptavidin conjugate (Invitrogen) by incubating a reaction containing 2.5 µM biotinylated CC-handle and 500 nM Qdot 705 at room temperature for 10 min. Since cohesin used in the experiments was also biotinylated it was blocked with excessive amount of Neutravidin by incubating a reaction containing 250 nM cohesin and 7.5 µM Neutravidin at room temperature for 10 min. Subsequently, 2 µM Qdot-labelled CC-handle and 100 nM Neutravidin-coupled cohesin were mixed and incubated at room temperature for 10 min. After that, 40 uL of the resulting complex was introduced into the flow-cell at 0.3 nM cohesin concentration in buffer L supplemented with 3 nM NIPBL at 15 uL/min and incubated for 5 min. The flow cell was further washed with 50 uL of buffer L supplemented with 3 nM NIPBL at 15 uL/min to remove unbound cohesin. DNA stained with Sytox Orange and cohesin coupled to CC-Qdot were visualised using a custom-built HILO microscopy set-up utilizing 488 nm and 561 nm lasers and a Nikon SR HP Apo TIRF 100x/1.49 oil immersion objective at 1Hz acquisition frame rate and 100ms exposure time. Images of the Sytox Orange and Qdot-705 channels were collected with two Andor Sona sCMOS camera through the Andor Optosplit II system. Collected images were saved as uncompressed TIFF files and further processed using FIJI ImageJ. We reliably observed DNA molecules where Qdot localised at the stem of the DNA loop, confirming that the CC handle-coupled cohesin retains DNA loop extrusion activity (Supplementary Fig. 7d).

SCC1 cleavage assessment

To test the efficiency of SCC1 cleavage we utilized the following experiment. The flow cell was coated with anti-Digoxigenin antibodies and passivated as in the protocol for DNA loop extrusion assay (see above). To obtain DNA stretched on the surface 150 µl of 50 pM of Digoxigenin-labelled λ-phage DNA in buffer D were introduced into the flow cell at 15 µl/min and immediately washed with 150 µl of buffer D at 15 µl/min.

Biotinylated cohesin was coupled to a Qdot 705 streptavidin conjugate (Invitrogen) for 5 min at room temperature in a reaction containing 250 nM cohesin and 500 nM Qdot 705. Next, 40 uL of 1 nM QDot-coupled cohesin in Binding buffer (Tris-HCl pH 7.5 40 mM, 50 mM NaCl, 5 mM DTT, β-Casein 1mg/mL) were introduced into the flow cell at 10 uL/min and incubated for 5 min. The flow cell was washed with 100 uL of High-salt buffer (Tris-HCl pH 7.5 40 mM, 600 mM NaCl, 5 mM DTT, β-Casein 1mg/mL) at 20 uL/min. DNA bound cohesin resisting high salt concentration was visualised with a custom-built HILO microscopy set-up utilizing a 488 nm laser and a Nikon SR HP Apo TIRF 100x/1.49 oil immersion objective at 5Hz acquisition frame rate and 100ms exposure time. Images were collected with Andor Sona sCMOS camera, saved as uncompressed TIFF files and further processed using FIJI ImageJ. Then, the high salt was washed out, and SCC1 cleavage was done by flowing in 40 uL of TEV protease (NEB) diluted 15X in TEV buffer (Tris-HCl 40 mM, DTT 1 mM) and incubating for 10 min. Next, the flow cell was washed with 50 uL of High-salt buffer and remaining cohesin was visualized at 5Hz. To estimate the efficiency of the kleisin cleavage we analysed 10 fields of view with cohesin molecules rapidly moving along DNA before and after TEV treatment. Out of 66 cohesin molecules rapidly translocating on DNA before addition of TEV protease, 56 molecules (85%) were released and only 10 molecules (15%) remained translocating on DNA after TEV incubation, which confirmed the efficient SCC1 cleavage (Supplementary Fig. 2b,c). All experiments were performed at room temperature.

References

1. Higashi, T. L., Pobegalov, G., Tang, M., Molodtsov, M. I. & Uhlmann, F. A Brownian ratchet model for DNA loop extrusion by the cohesin complex. *Elife* **10**, (2021).

Supplementary Figures

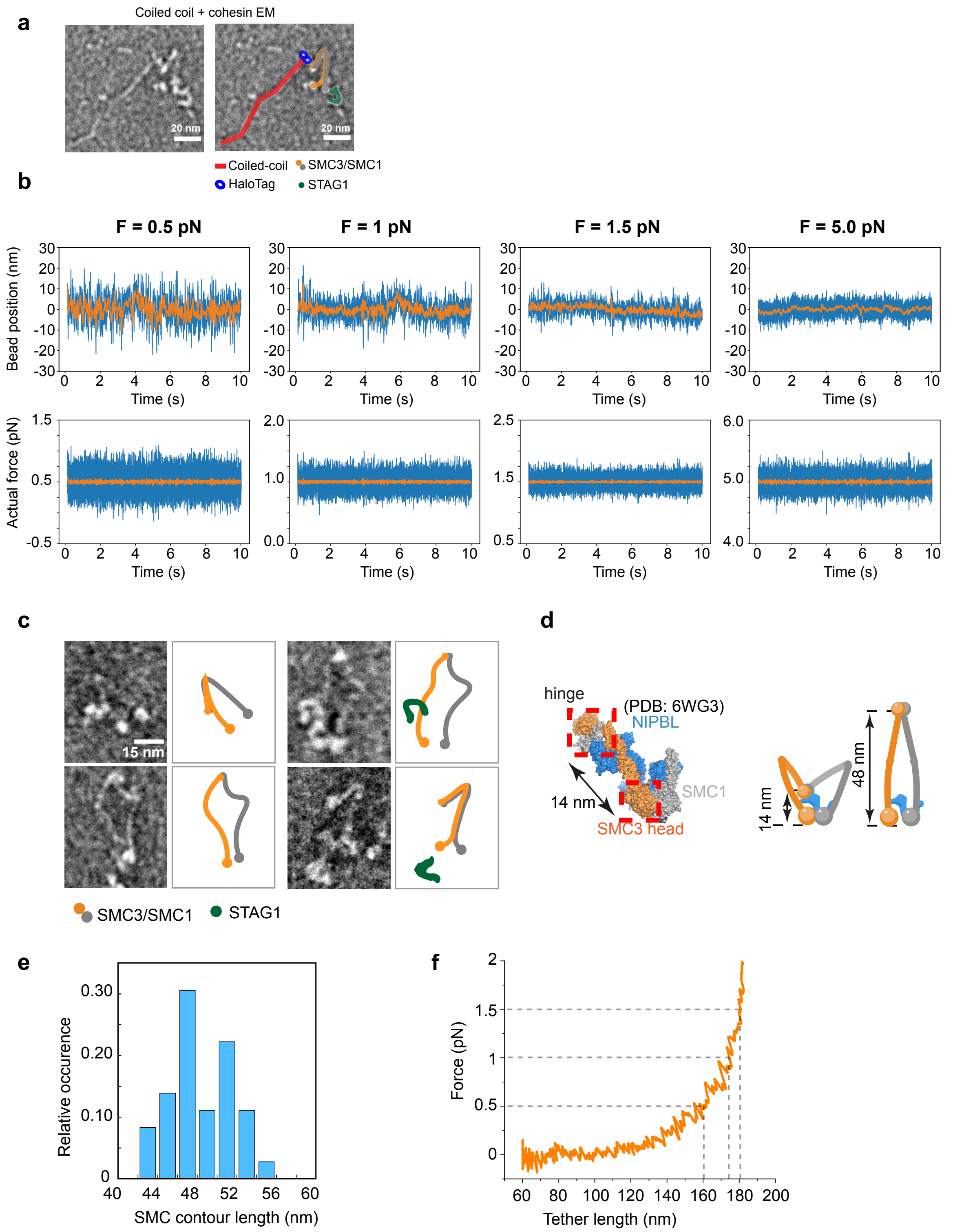

Supplementary Fig. 1 | Additional characterization of the assay. a Negative staining EM image of human cohesin coupled to a coiled-coil handle via the hinge domain labelled with HaloTag-ligand. **b** Top: Positions of a bead attached to an inactive cohesin complex under tension in force-clamped experiments sampled at 2kHz (blue) and downsampled to 50Hz by moving average (orange). Target force is shown on top. Bottom: Corresponding force traces

showing actual measured forces. **c** Negative staining EM images of human cohesin (representative images of total 36 cohesin molecules). **d** Calculation of the minimum and maximum head-hinge distance of the human cohesin based on cryo-EM structure (PDB, 6WG3) and SMC contour length measurement. **e** Distribution of the SMC contour length derived from negative staining EM of individual human cohesin molecules as shown in **a** (n=36). **f** Force extension curve of the head-hinge cohesin tether (cohesin plus coiled-coil linker) in the low force regime.

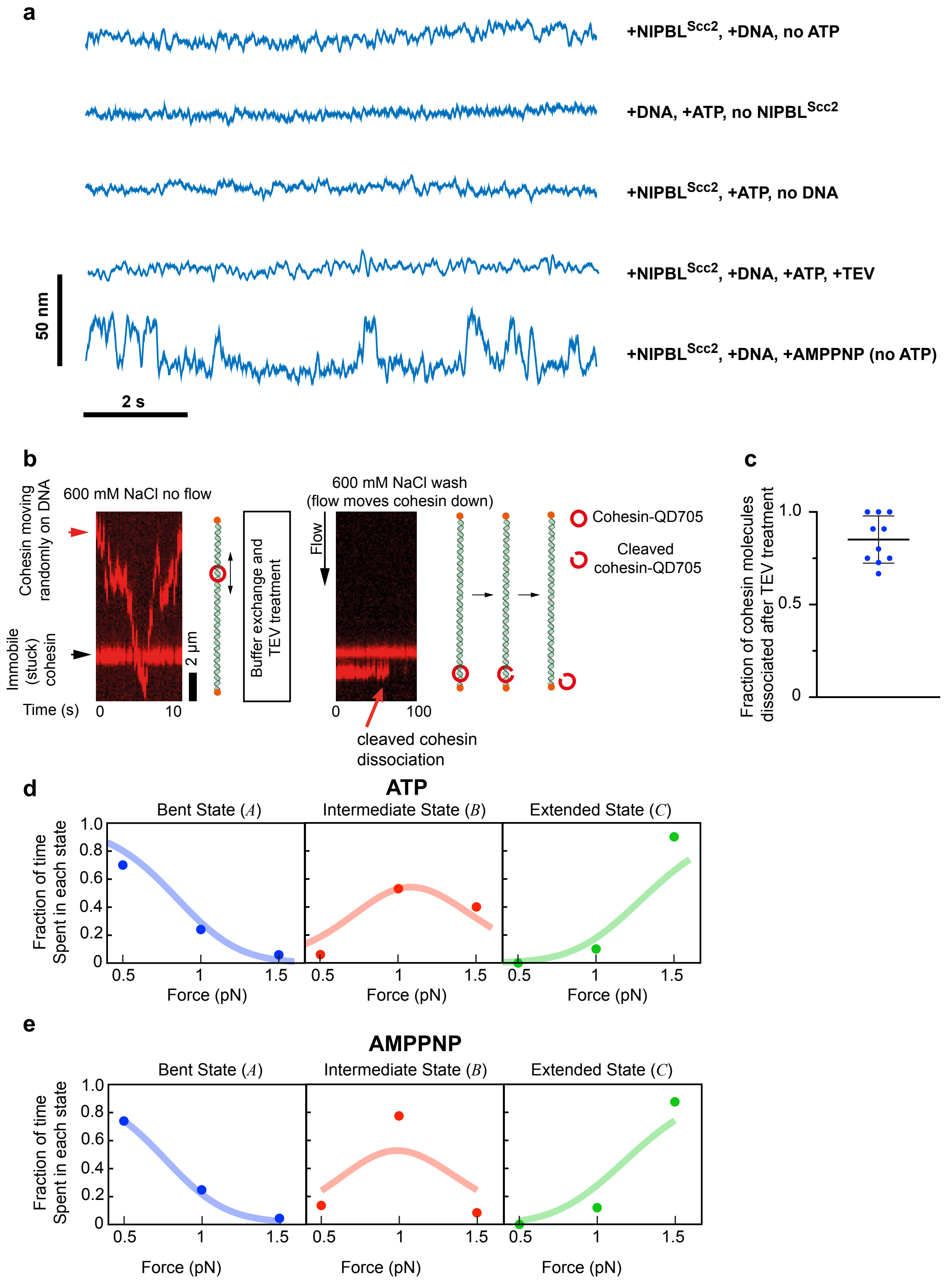

Supplementary Fig. 2 | Quantification of the cohesin head-hinge movement. a Typical examples of traces for the relative head-hinge distance under 1 pN tension for different experimental conditions shown on the right. All traces are scaled the same way and scale bars are shown on the left. **b** An example kymograph that shows high-salt resistant cohesin moving on DNA being removed after the TEV treatment. The kymograph is split into two pieces separated by the TEV treatment. **c** Quantification of the events from **b** shows fractions

of salt-resistant cohesin molecules that were removed from DNA after the TEV treatment (N = 10 fields of view; line: mean; error bars: standard deviation). **d** Fit of the data from Fig. 1d to the three-state model on Fig. 1f. Dots show relative time spend in each state for different forces and solid lines show relative probabilities of finding the system in one of the three states as derived in methods. The fit parameters (See Fig. 1f) are $K_0^{1,2}$=0.03 (0.005-0.14), $K_0^{2,3}$=0.01 (0.003-0.06). Values in brackets show 80% confidence interval. **e** Same as in **d**, but for the condition in which ATP was replaced with AMPPNP. The fit parameters are $K_0^{1,2}$=0.04 (0.006-0.2), $K_0^{2,3}$=0.01 (0.002-0.04).

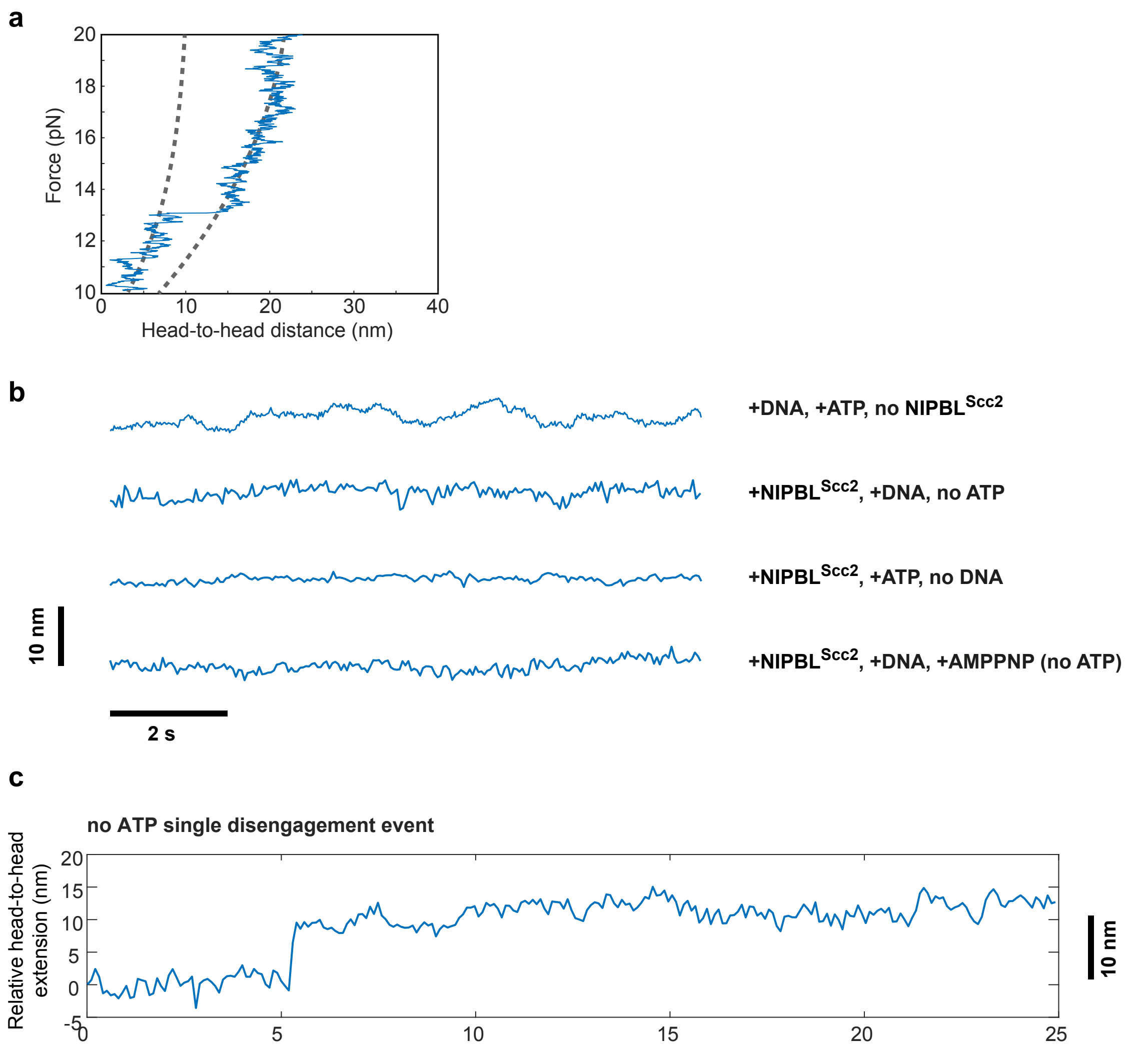

Supplementary Fig. 3 | Quantification of the cohesin head-head movement. a An example of a force-extension curve for the cohesin head-head construct in the high force regime. The curve shows single disengagement event that occurred during the measurement at ~ 13 pN force. **b** Typical examples of traces for the relative head-head distance under external force for different experimental conditions shown on the right. All traces are scaled the same way and scale bars are shown on the left. **c** An example trace for the head-head construct under 10 pN force obtained without ATP that shows single disengagement event not followed by engagement.

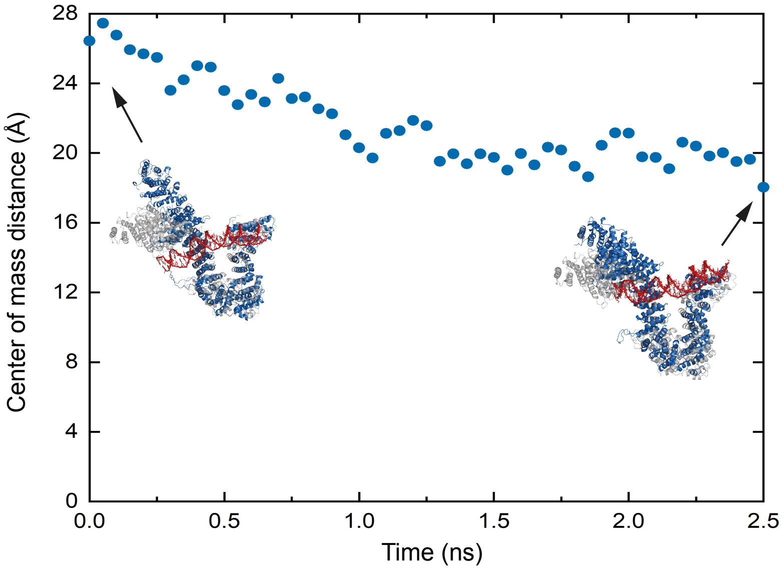

Supplementary Fig. 4 | Equilibration of the "relaxed" NIPBL^{Scc2} with DNA. The process of equilibration of the constructed model of "relaxed" NIPBL with DNA during which the center of mass of the N terminus (See Fig. 3b) is changing and the final equilibrated NIPBL^{Scc2} with DNA becomes slightly bent. Blue structures show NIPBL^{Scc2} with DNA before (left) and after (right) equilibration. NIPBL^{Scc2} shown in grey is the "bent" structure shown for comparison.

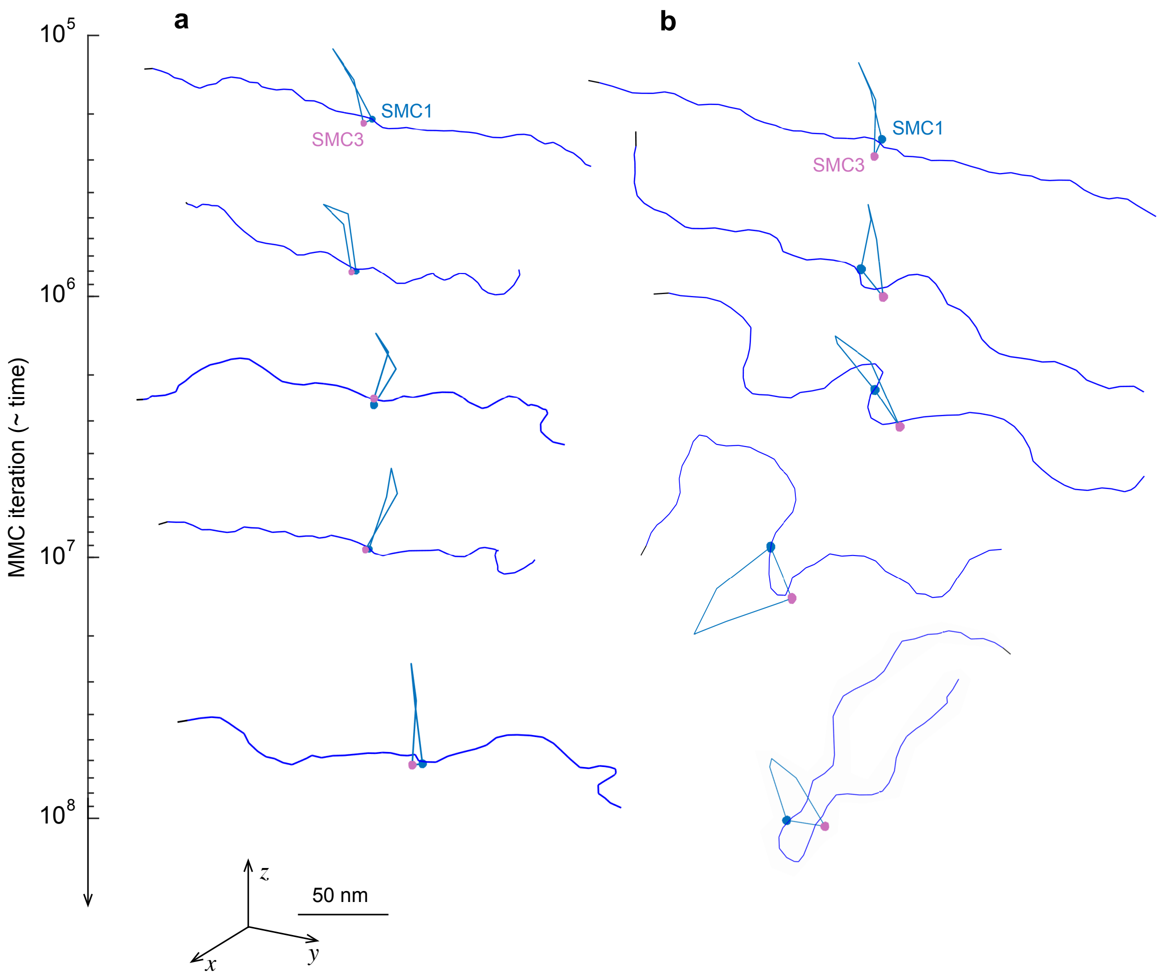

Supplementary Fig. 5 | Metropolis Monte-Carlo model of cohesin head disengagement driving initiation of DNA loops. a and **b** show still frames from simulations in which head disengagement either cannot (**a**) or can (**b**) generate force. The system was initialized in the head engaged state. SMC1 was allowed to bind to DNA and then the state was changed to the equilibrium corresponding to disengaged heads. As heads disengage SMC1 keeps contact with DNA, while SMC3 allows DNA sliding. **a** When heads cannot push against each other, they cannot disengage because both are bound to DNA very close. **b** When disengagement can generate force, the force leads to DNA bending and head disengagement. Cohesin is light blue and SMC3 is pink for clarity. These simulations did not consider head-hinge movement and the SMC coiled coils were assumed to be in their extended state.

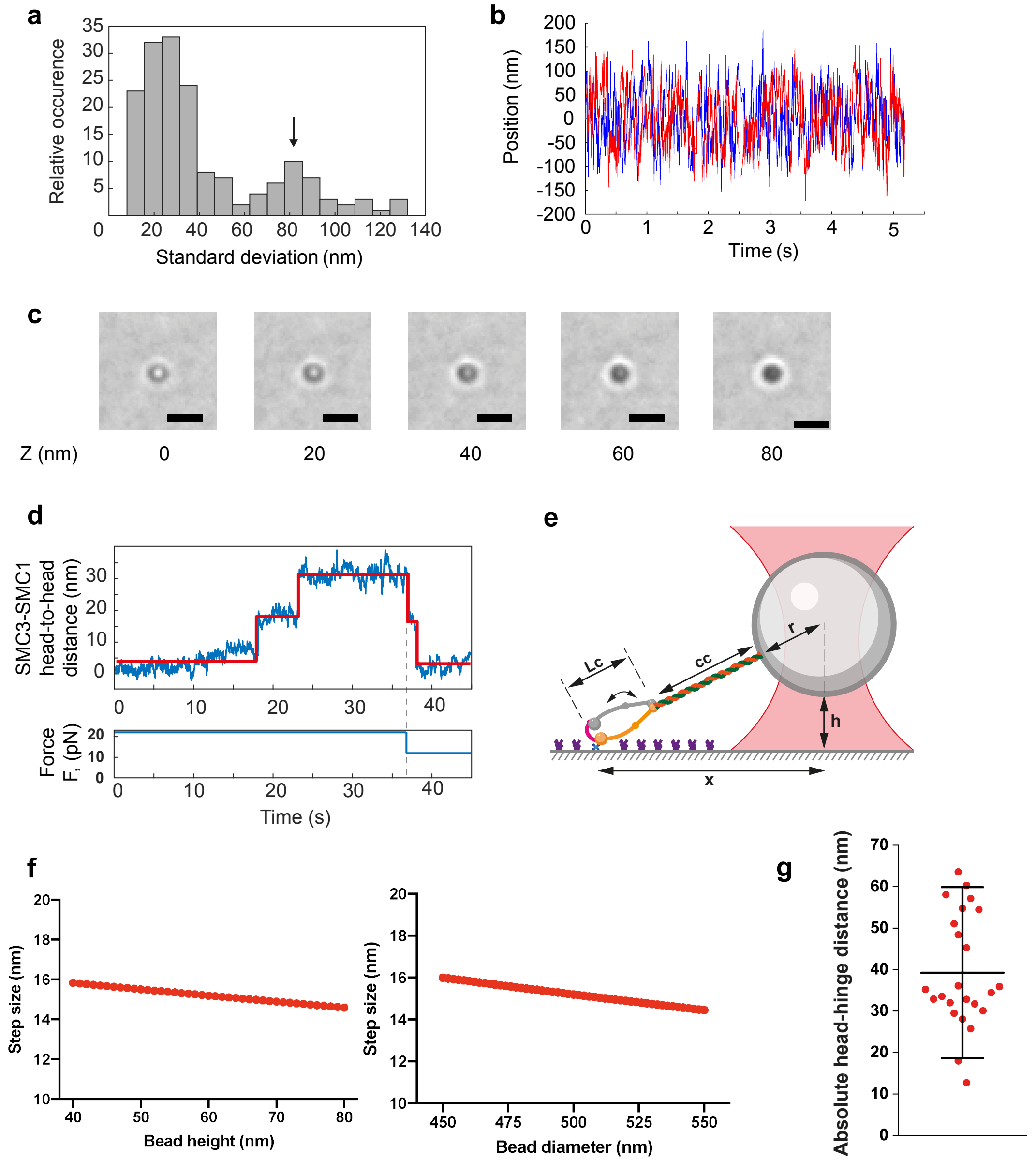

Supplementary Fig. 6 | Data processing and analysis used in the assay. a Distribution of the standard deviations of bead positions extracted from tethered particle motion experiments by video analysis. An arrow points to the peak formed by single tethers. **b** Blue and red show *x* and *y* traces of the bead position tethered to a surface via single cohesin and a myosin handle in the absence of the applied force extracted by video tracking position of the bead. **c** Images of the 500 nm size bead used in the optical trapping experiments at different axial positions of the Z-stage after it touches the coverslip, which pushes it axially out of the trap. The position of the stage is shown under each image. Scale bar is 1 micron (representative images of more than 20 independent experiments). **d** Example trace of an experiment in which high force (>20pN) was applied to cohesin head-head interface leading to the two-step extension. As the force reduced to 10 pN, cohesin immediately compacts. After a delay another step occurs. The amplitude of the second step is 10 nm and it likely corresponds to the head engagement. **e** Schematic of the cohesin length calculation in the optical tweezers assay: Lc – cohesin length, CC – length of the coiled-coil handle (110 nm), r – bead radius

(250 nm), h – bead height (60 nm), x – piezo stage displacement. **f** A step size of 15 nm from a typical recording of the head-hinge cohesin at 1 pN transitioning between the intermediate and extended states was calculated for different bead axial distance from the coverslip (bead height) and bead diameter. **g** Absolute head-hinge distance determined experimentally for a cohesin head-hinge construct stretched at 1.5 pN (n = 24 molecules, line: mean, error bars: standard deviation).

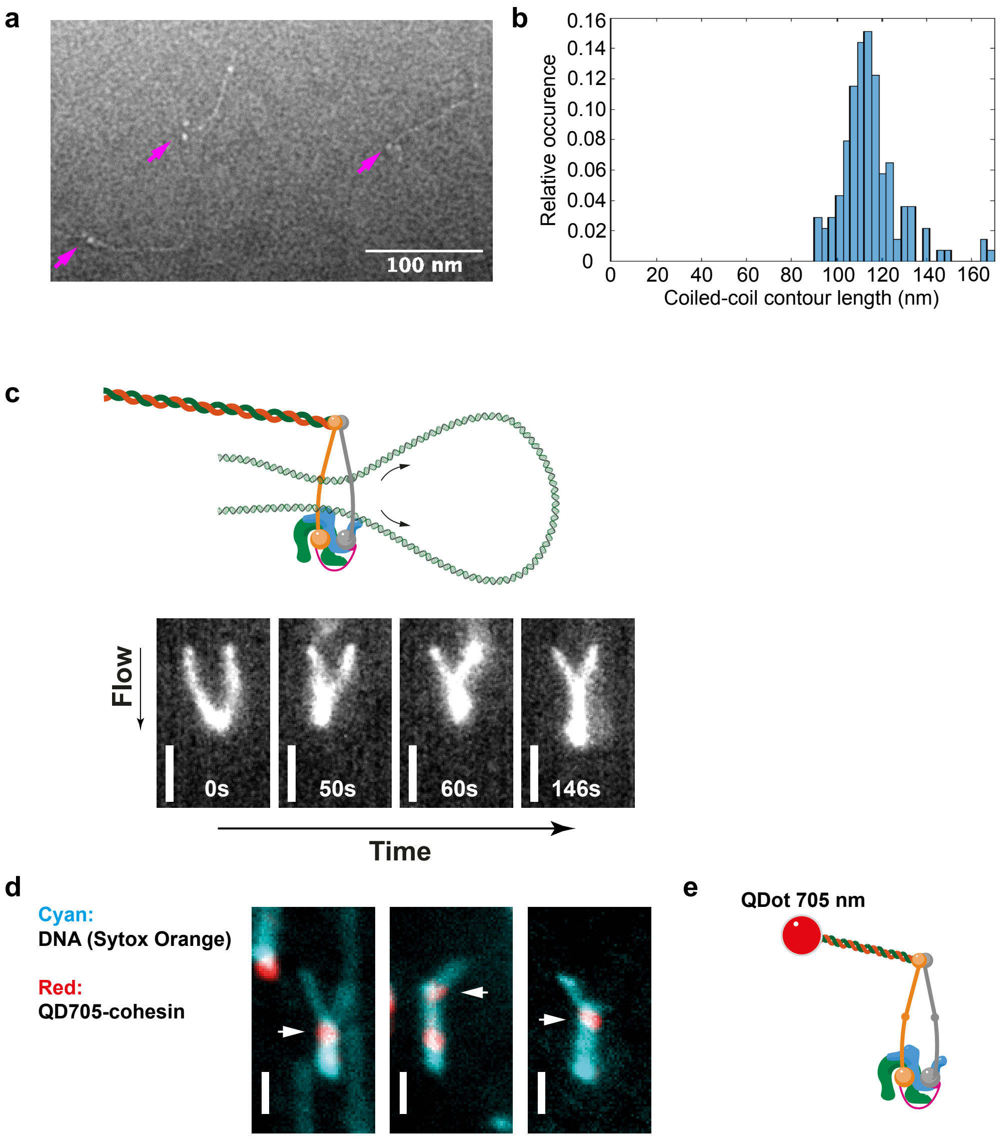

Supplementary Fig. 7 | Characterization of the passive coiled-coil linker. a Negative stain example image of the coiled-coil handle possessing two HaloTags at one terminus and biotinylated at the other. Magenta arrows indicate HaloTags (representative images of total 139 molecules). **b** Distribution of the coiled-coil handle lengths measured from multiple images. **c** Example of DNA loop extrusion by human cohesin coupled to a coiled-coil handle in the presence of NIPBL^{Scc2} and ATP. Lambda DNA (48.5 kbp) is tethered to the surface, stretched with a flow and visualized by staining with Sytox Orange (representative images of more than 10 independent experiments). **d** Examples of DNA loops extruded by cohesin complex coupled to the coiled coil. The complex was visualized by attaching a quantum dot to the end of the coiled coil linker distant from cohesin. Quantum dot (QDot705) is shown in red. DNA stained with Sytox Orange is in cyan. Scale bar is 2 μm (representative images of 4 independent experiments). **e** Schematics of the complex used in DNA loop extrusion experiment in **d**.
