# Supplementary material for: Single cohesin molecules generate force by two distinct mechanisms
Source: Nat Commun. 2023 Jul 4;14:3946. doi: 10.1038/s41467-023-39696-8 (PMC10319895; doi:10.1038/s41467-023-39696-8)
Supplement: Supplementary file 3 — Reporting Summary [file 41467_2023_39696_MOESM3_ESM.pdf]

nature portfolio

Corresponding author(s): Maxim Molodtsov
Last updated by author(s): May 25, 2023

Reporting Summary

Nature Portfolio wishes to improve the reproducibility of the work that we publish. This form provides structure for consistency and transparency in reporting. For further information on Nature Portfolio policies, see our Editorial Policies and the Editorial Policy Checklist.

Statistics

For all statistical analyses, confirm that the following items are present in the figure legend, table legend, main text, or Methods section.

n/a	Confirmed	
☐	☒	The exact sample size (*n*) for each experimental group/condition, given as a discrete number and unit of measurement
☐	☒	A statement on whether measurements were taken from distinct samples or whether the same sample was measured repeatedly
☒	☐	The statistical test(s) used AND whether they are one- or two-sided<br>*Only common tests should be described solely by name; describe more complex techniques in the Methods section.*
☒	☐	A description of all covariates tested
☒	☐	A description of any assumptions or corrections, such as tests of normality and adjustment for multiple comparisons
☐	☒	A full description of the statistical parameters including central tendency (e.g. means) or other basic estimates (e.g. regression coefficient) AND variation (e.g. standard deviation) or associated estimates of uncertainty (e.g. confidence intervals)
☒	☐	For null hypothesis testing, the test statistic (e.g. *F*, *t*, *r*) with confidence intervals, effect sizes, degrees of freedom and *P* value noted<br>*Give P values as exact values whenever suitable.*
☐	☒	For Bayesian analysis, information on the choice of priors and Markov chain Monte Carlo settings
☒	☐	For hierarchical and complex designs, identification of the appropriate level for tests and full reporting of outcomes
☒	☐	Estimates of effect sizes (e.g. Cohen's *d*, Pearson's *r*), indicating how they were calculated

Our web collection on statistics for biologists contains articles on many of the points above.

Software and code

Policy information about availability of computer code

Data collection	For optical tweezers calibration, operation and measurements we used JPK NT software package.<br>For acquisition of fluorescence images we used JPK NT software package and Andor Solis software.<br>For MD simulations we used VMD, GROMACS 2019, PLUMED 2.6.6.
Data analysis	We used JPK Data Processing Software package for optical tweezers data extraction;<br>MatLab R2020a and OriginLab Pro 2021 for optical tweezers data analysis;<br>Fiji ImageJ 2.3.0/1.53q to analyze fluorescence data and EM micrographs.<br>Final figures were prepared using Adobe Illustrator 2020.<br>Codes for Monte-Carlo simulations (MatLab) and MD simulations (GROMACS and PLUMED) are available at https://github.com/FrancisCrickInstitute/CohesinModel.

For manuscripts utilizing custom algorithms or software that are central to the research but not yet described in published literature, software must be made available to editors and reviewers. We strongly encourage code deposition in a community repository (e.g. GitHub). See the Nature Portfolio guidelines for submitting code & software for further information.

Data

Policy information about availability of data

All manuscripts must include a data availability statement. This statement should provide the following information, where applicable:

- Accession codes, unique identifiers, or web links for publicly available datasets
- A description of any restrictions on data availability
- For clinical datasets or third party data, please ensure that the statement adheres to our policy

Source data files are provided with this paper. Raw data will be made available by the authors upon request. MD simulations used PDB data with PDBIDs: 6WG3, 6YUF, 6ZZ6 and 5T8V. The simulation code with a sample is available at: https://github.com/FrancisCrickInstitute/CohesinModel

Human research participants

Policy information about studies involving human research participants and Sex and Gender in Research.

Reporting on sex and gender	N/A
Population characteristics	N/A
Recruitment	N/A
Ethics oversight	N/A

Note that full information on the approval of the study protocol must also be provided in the manuscript.

Field-specific reporting

Please select the one below that is the best fit for your research. If you are not sure, read the appropriate sections before making your selection.

☒ Life sciences ☐ Behavioural & social sciences ☐ Ecological, evolutionary & environmental sciences

For a reference copy of the document with all sections, see nature.com/documents/nr-reporting-summary-flat.pdf

Life sciences study design

All studies must disclose on these points even when the disclosure is negative.

Sample size	Sample size was not predetermined. Data was collected until statistical significance was reached. Sample size for the force measurement is conditioned by the intrinsic low throughput of single-molecule optical trapping experiments, which limits the number of measured molecules to typically 3-5 molecules on a successful experimental day. All observations of force-generation by cohesin molecules are based on at least 15 molecules measured per each condition in several independent experiments. This sample size was sufficient to determine the experimentally measured parameters with accuracy that supported conclusions of this work. We indicated the precise sample size for each experiment in the figure legends and in the text.
Data exclusions	For three-state analysis of the head-hinge movement we selected only cohesin molecules that could be clearly attributed to at least two of the three states, i.e. exhibited transitions between fully bent and fully extended conformations resulting in a ~ 32 nm change of the tether length. Static tethers and tetheres showing only a ~ 15 nm change in length were not included into the three-state analysis because the conformational state of such molecules could not be determined. This exclusion criteria were pre-established.
Replication	All the experiments were repeated at least three times on separate measurement days with freshly prepared buffers and samples. All reported data were reproducible.
Randomization	Single-molecule data with the same conditions is inherently randomized and no other special randomization was implemented.
Blinding	Step detection and size extraction in single-molecule experiments employed in this work are not affected by the knowledge or beliefs of the investigator, and, therefore, blinding was not used. To prevent other biases, both the first author and last authors separately and independently performed data collection and analysis using procedures described in Methods.

Reporting for specific materials, systems and methods

We require information from authors about some types of materials, experimental systems and methods used in many studies. Here, indicate whether each material, system or method listed is relevant to your study. If you are not sure if a list item applies to your research, read the appropriate section before selecting a response.

Materials & experimental systems

n/a	Involved in the study
☐	☒ Antibodies
☒	☐ Eukaryotic cell lines
☒	☐ Palaeontology and archaeology
☒	☐ Animals and other organisms
☒	☐ Clinical data
☒	☐ Dual use research of concern

Methods

n/a	Involved in the study
☒	☐ ChIP-seq
☒	☐ Flow cytometry
☒	☐ MRI-based neuroimaging

Antibodies

Antibodies used

Anti-Digoxigenin-AP, Fab fragments. Roche, 11093274910.

Validation

This commercially available antibody was validated by the manufacturer, Roche, for the detection of digoxigenin-labeled compounds, such as Digoxigenin-labeled nucleic acids. In this work, antibodies were absorbed on the surface of a microfluidic flow-cell for the attachment of Digoxigenin-labeled DNA molecules. The attachment was confirmed by imaging tethered DNA molecules using fluorescence microscopy. This experiment provided the required validation.
